# Evaluation on curative effects of gabapentin for the prevention of chronic pain in adults following surgery: A protocol for systematic review and meta-analysis

**DOI:** 10.1097/MD.0000000000027558

**Published:** 2021-10-22

**Authors:** Yao Chen, Guang-Zhi Zhang

**Affiliations:** a Edong Healthcare City Hospital of Traditional Chinese Medicine (Infectious Disease Hospital); Ezhou, Hubei, China; b Department of Pain Management, People's Hospital of Dongxihu District, Wuhan, Hubei, China.

**Keywords:** efficacy, gabapentin, meta, pain, surgery

## Abstract

**Background:**

Chronic pain is a common postsurgery symptom. It has an adverse impact on patients’ overall wellbeing. Chronic pain after an operation is caused by intricate mechanisms that have not been well understood. The predictability of most surgical methods has enabled scholars to conduct randomized controlled trials (RCTs) involving pharmacological interventions to prevent chronic pain after a surgery. Therefore, the present study aims to evaluate the curative effects of gabapentin to prevent chronic pain in adults after surgery.

**Methods:**

The authors will collect RCTs related to the use of gabapentin to prevent chronic pain in adults following surgery. Accordingly, a comprehensive search will be performed in 4 online databases to find English language articles, including Cochrane Library, EMBASE, Web of Science, and PubMed. In addition, the search also includes 3 Chinese language databases: VIP data, WanFang database, and China National Knowledge Infrastructure. Each of the RCT published from their inception to September 2021 will be considered. The authors will carry out a meta-analysis of RCTs after screening the studies. Subsequently, the authors will use RevMan (v 5.3) to perform an assessment of bias risk, data synthesis, and subgroup analysis, provided inclusion criteria are met.

**Results:**

The results will provide clinical evidence for the curative effects of gabapentin to prevent chronic pain in adults after surgery.

**Conclusion:**

The summary provided in this systematic review will judge whether gabapentin intervention is effective and feasible to prevent chronic pain in adults following surgery.

**Registration number:** 10.17605/OSF.IO/XG4CK

## 1 Introduction

Chronic postsurgical pain (CPSP) alludes to a disabling complication that could severely impact a patient's wellbeing and quality of life. CPSP results in pain that could at times last for extensive periods postsurgery. Generally, 10% of patients who undergo common surgical treatment will end up suffering from chronic pain.^[1–3]^ According to definition, CPSP is a pathological pain persistent for over 2 months postsurgery.^[4–6]^ Given the difficulty in managing CPSP, many efforts have been undertaken to stop the shift from severe pain to chronic pain, such as administering numerous systemic pharmacologic intercessions before the surgery.^[7]^

Many adopt multimodal strategies to expand perioperative pain control, and these advances have allowed clinical practitioners to benefit from the additive or synergistic effects of numerous analgesics while lessening related side effects.^[8–10]^ Pre-emptive analgesia is another common means to avert central and peripheral sensitization in pain, despite the absence of a standard effective regimen.^[11,12]^ Reportedly, using gabapentinoids as adjunct analgesics before a surgery induces an opioid-sparing effect, and a reduction in side effects from opioids, such as vomiting and postoperative nausea, as well as lesser pain scores.^[13,14]^

This study aims to synthesize evidence in randomized controlled trials (RCTs) related to the efficacy and safeness associated with using gabapentin to prevent the progression of CPSP.

## 2 Methods

This review is registered under the Open Science Framework (OSF) (registration number, 10.17605/OSF.IO/XG4CK) on September, 2021. This protocol will be reported according to the Preferred Reporting Items for Systematic Review and Meta-Analysis Protocols (PRISMA-P) 2015 statement.^[15]^

## 3 Eligibility criteria

### 3.1 Types of study

RCTs that explored the efficacy and security of using gabapentin to prevent chronic pain in adults will be included in this study.

### 3.2 Types of participants

Adults (aged 18 and above) due for surgical treatments for tissue injuries.

### 3.3 Types of interventions

All administered drugs in the immediate period presurgery, during the surgery, or postsurgery, by any dosage, frequency, or method.

### 3.4 Types of outcome measures

The percentage of patients who indicate all forms of pain in the vicinity of the anatomical site or pain related to the site of surgery, or both, over 3 months after the operation are the primary outcomes of this study.^[2]^ The secondary outcomes include the number of patients who report moderate to extreme pain at the anatomical site of the surgery or pain referring to the surgical site-or both-6 months after the operation, and how many participants dropped out from the examination because of adverse effects from the treatment.

## 4 Search methods for identification of studies

### 4.1 Electronic searches

The authors will collect RCTs related to the use of gabapentin to prevent chronic pain in adults following surgery. Accordingly, a comprehensive search will be performed in 4 online databases to find English language articles, including Cochrane Library, EMBASE, Web of Science, and PubMed. In addition, the search also includes 3 Chinese language databases: VIP data, WanFang database, and China National Knowledge Infrastructure. Each of the RCT published from their inception to September 2021 will be considered. Search terms include pain, gabapentin, and RCTs.

### 4.2 Searching other resources

We will use search engines to search related literature on the Internet, these include Google Scholar and Baidu Academic. Moreover, the authors will search ClinicalTrials.gov (https://clinicaltrials.gov/) to obtain related clinical studies. Besides, if necessary, we will contact investigators for relevant results. Additionally, we will manually perform citation searches to avoid missing critical information.

## 5 Data collection and analysis

### 5.1 Selection of studies

Two reviewers will sift the search results and identified number of RCTs to include in the review.

### 5.2 Data extraction and management

The authors will extract data from each study, such as study drug name(s), dosages(s), method(s), timing and duration, operating technique, and percentage of patients: reporting pain after a period of 3 months or more following the surgery, reporting a minimum 4/10 or average to extreme pain in 3 months or more postsurgery, and the percentage of patients who drop out of the study due to adverse effects from treatment. A couple of autonomous authors will conduct data extraction. Accordingly, they will read each eligible study and complete the extraction of data from each eligible article.

### 5.3 Assessment of risk of bias in included studies

The bias risk associated with each eligible study was graded according to the Cochrane risk of bias tool.^[16]^

### 5.4 Measures of treatment effect

To represent dichotomous outcomes, this study will use the relative risk and for continuous data, the authors will calculate the mean differences between treatment groups when articles report identical results. If identical results are reported on different scales, we will compute the mean differences or standardized mean differences. All analyses will consider the 95% confidence interval.

### 5.5 Assessment of heterogeneity

A heterogeneity test will be employed to assess the heterogeneity, which is expressed by *I*^2^ value. If *I*^2^ < 25%, we will consider it to be small heterogeneity. If 25% < *I*^2^ < 50%, we will consider it to be moderate heterogeneity. If *I*^2^ ≥ 50%, we will consider it to be large heterogeneity.

### 5.6 Assessment of reporting bias

The authors will evaluate the reporting biases, including publication bias and use funnel plots provided the number of eligible studies exceed 10. In the case where a reporting bias is signified by the funnel plot's asymmetry, the authors will attempt to explain it.

### 5.7 Sensitivity analysis

The authors will perform a sensitivity analyses to determine how robust the meta-analysis results are. It will be performed by excluding articles having high bias risk and numerically distant outliers from the remainder of the data.

## 6 Ethics and dissemination

This protocol does not need an ethics approval because it is a systematic review that uses already published information.

## 7 Discussion

The proposed protocol for a meta-analysis shall be carried out to evaluate the efficacy and security of using gabapentin to prevent chronic pain in adult patients after surgical treatment. Currently, there has been no systematic review and meta-analysis on the topic. Thus, it is critical and extremely relevant to conduct this study to further investigate the efficacy and security of using gabapentin to prevent chronic pain in adults after surgery. We will retrieve all related literature without language restrictions in the current systematic review. All studies involving the use of gabapentin for CPSP shall be considered to prevent excluding potential trials. The results of this meta-analysis will summarize current reports on the efficacy and security of using gabapentin to stop chronic pain from impacting adult patients after surgery. The evidence could also yield helpful evidence for clinical practice and those who devise health policies.

## Author contributions

**Conceptualization:** Yao Chen, Guang-Zhi Zhang.

**Data curation:** Yao Chen, Guang-Zhi Zhang.

**Formal analysis:** Yao Chen.

**Funding acquisition:** Guang-Zhi Zhang.

**Investigation:** Yao Chen, Guang-Zhi Zhang.

**Methodology:** Yao Chen, Guang-Zhi Zhang.

**Project administration:** Guang-Zhi Zhang.

**Resources:** Yao Chen.

**Software:** Yao Chen, Guang-Zhi Zhang.

**Supervision:** Guang-Zhi Zhang.

**Validation:** Yao Chen, Guang-Zhi Zhang.

**Visualization:** Yao Chen, Guang-Zhi Zhang.

**Writing – original draft:** Yao Chen, Guang-Zhi Zhang.

**Writing – review & editing:** Guang-Zhi Zhang.
